# Right ventricular exclusion for hepatocellular carcinoma metastatic to the heart

**DOI:** 10.1186/1749-8090-5-95

**Published:** 2010-10-30

**Authors:** Wan-Chi Liu, Kar-Wei Lui, Ming-Chi Ho, Shou-Zen Fan, Anne Chao

**Affiliations:** 1 Department of Anesthesiology, National Taiwan University Hospital, Taipei, Taiwan; 2 Department of Diagnostic Radiology, Chang Gung Memorial Hospital, Taoyuan, Taiwan; 3 Department of Surgery, National Taiwan University Hospital, Taipei, Taiwan

## Abstract

We used for the first time a right ventricular exclusion procedure for the treatment of hepatocellular carcinoma metastatic to the right ventricle. Our case report shows that this surgical option can be effective as rescue therapy for right ventricular outflow tract obstruction secondary to myocardial metastasis in critically ill patients. Most notably, this technique can prevent inadvertent dislodgement of tumor cells.

## Background

Right ventricular outflow tract obstruction secondary to myocardial metastasis from hepatocellular carcinoma (HCC) represents a rare event and portends a poor prognosis [1-4]. The clinical picture is chiefly dominated by severe cardiorespiratory compromise that may lead to cardiac arrest. Surgical resection with therapeutic intent is not an option for the majority of patients with metastatic involvement of the heart. However, symptom relief after palliative surgery can improve quality of life. We hereby present a clinical case of intraventricular cardiac metastasis from HCC leading to right ventricular outflow tract obstruction. We used for the first time a right ventricular exclusion procedure as rescue therapy to relieve mechanical obstruction to blood flow and avoid life-threatening hemodynamic instability. In addition, this procedure can prevent inadvertent dislodgement of tumor cells.

## Case Presentation

A 46-year-old female patient complained of general weakness and increasing dyspnea for 1 month. She had been diagnosed 14 months earlier with a hepatocellular carcinoma for which she underwent extended right hepatectomy. After surgery, the patient was treated twice with transarterial chemoembolization for small recurrent HCC lesions. At the time of the second chemoembolization, computed tomography (CT) and magnetic resonance imaging (MRI) revealed a right ventricular mass resulting in right ventricular outflow tract obstruction. The patient was offered surgery but, being otherwise asymptomatic, she refused treatment at that time.

**Figure 1:**
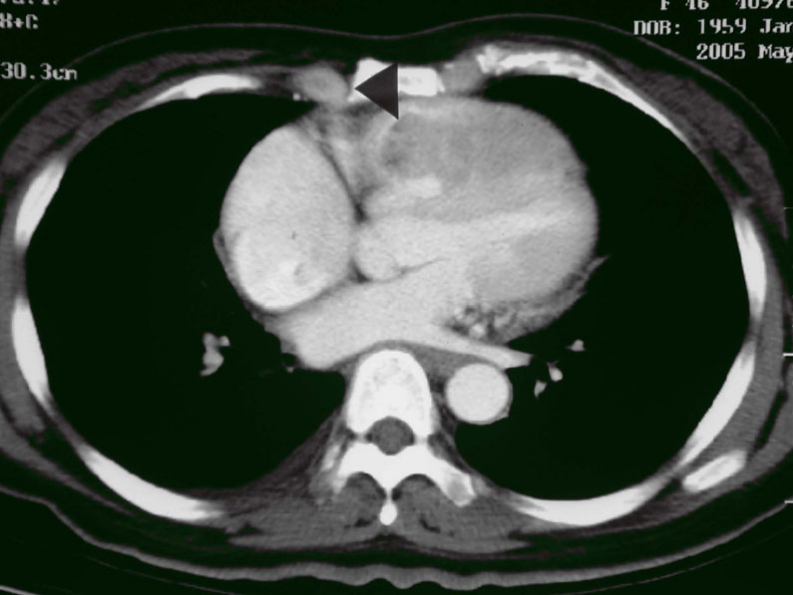
**Computed tomography showing a mildly enhancing mass (arrowhead) surrounded by contrast medium**. The mass was attached to the right ventricular wall, extending to the main pulmonary artery.

**Figure 2:**
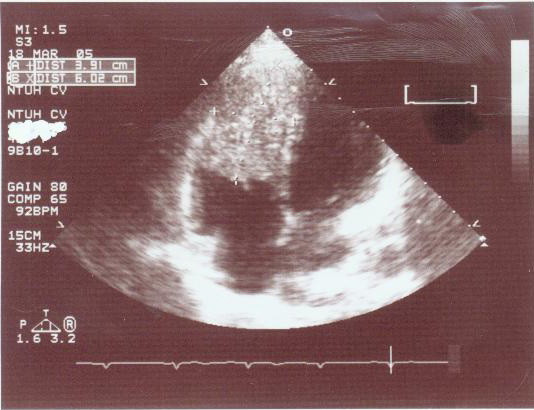
**Echocardiography showing a mass occupying most of the right ventricle**.

Two months after hospital discharge, she developed marked exercise intolerance, dyspnea, and orthopnea. The patient was admitted for further investigation. Her pulse rate was 120 beats per minute, blood pressure 90/45 mmHg, and respiratory rate was 35 breaths per minute. A CT scan (Figure 1) and echocardiography (Figure 2) revealed a large tumor mass in the right ventricle extending to the right ventricular outflow tract and the proximal main pulmonary artery. The mass occasionally caused obstruction of the flow of blood through the tricuspid valve into the right ventricle. CT scan of abdomen showed no local recurrence of the liver tumor. The patient was operated upon urgently; a standard procedure was performed with moderate hypothermia, cardiopulmonary bypass, and bicaval cannulation. The heart was arrested with a cold blood cardioplegic solution administered intermittently. At surgery, a right ventriculotomy revealed a large cauliflower-like soft tissue mass of gray-yellow color invading right ventricular myocardium, the interventricular septum and septal papillary muscles. The right and left pulmonary arteries were temporarily occluded to prevent dislodging of tumor cells. Debulking of the mass was performed to relieve mechanical obstruction to blood flow, but the extensive infiltrating nature of the tumor prohibited complete removal. Owing to the incomplete resection, and because of the fragility of tumor surface after debulking, we reasoned that a right ventricular exclusion with total cavopulmonary connection (TCPC) could offer a viable approach with remarkable hemodynamic outcome while preventing dislodging. Therefore, the pulmonary and tricuspid valves were closed using a continuous suture, and the right ventriculotomy was closed with a patch. The superior vena cava was then transected and anastomosed to the upper aspect of the right pulmonary artery (RPA). An intracardiac conduit was constructed by using a GoreTex patch to direct inferior vena cava flow into the lower part of the RPA. A 6 mm fenestration was created to decompress the right side circulation. Cardiopulmonary bypass was weaned off smoothly and the immediate postoperative course was uneventful. The patient was extubated on the postoperative day 2, and she was transferred to ward on the postoperative day 6. Shortness of breath and tachypnea improved significantly after surgery. At oxygen flow rates of 3.0 L•min^-1^, a stable oxygen saturation ≥ 85% was reached. Pathological examination confirmed the diagnosis of metastatic HCC. The patient was subsequently placed on oral thalidomide maintenance therapy.

The patient experienced attacks of exertional dyspnea, and we performed transcatheter closure of the fenestration one month after TCPC. Arterial saturation improved significantly to 94% after fenestration closure, and exercise intolerance disappeared. Catheterization revealed a patent TCPC conduit. The patients refused to undergo the planned chemotherapy and radiotherapy for residual tumor in the right ventricle. She passed away four months after the surgery due to recurrence of HCC in liver.

## Discussion

Cases of HCC metastatic to the right ventricle are exceedingly rare and generally have a dismal prognosis [1-4]. There is only one report in the literature describing the use of cardiac surgery to remove a hepatocellular carcinoma that had metastasized to the right ventricle [4]. Management of metastasis to the heart is palliative surgical excision and this was followed in our patient by debulking of the mass to relieve mechanical obstruction to blood flow and avoid life-threatening hemodynamic instability. Most notably, the total right ventricular exclusion procedure used in our patient provides a means for avoiding tumor fragmentation, dislodgement, or embolization.

To improve a poor prognosis of metastatic HCC, multimodal approaches combining chemotherapy, radiotherapy, and surgery may be useful. Interestingly, it has been recently suggested that the oral multikinase inhibitor, sorafenib, may produce a survival advantage in patients with advanced HCC [5]. In conclusion, we used for the first time a right ventricular exclusion procedure for the treatment of HCC metastatic to the right ventricle. We believe that this surgical option can be effective as rescue therapy for right ventricular outflow tract obstruction secondary to myocardial metastasis in critically ill patients. Most notably, it can prevent inadvertent dislodgement of tumor cells.

## Competing interests

The authors declare that they have no competing interests.

## Authors' contributions

SZF and AC conceived of the study idea and participated in its designed. WCL and MCH participated in acquisition of patient data. WCL and MCH did mainly the literature review. KWL did image reading. WCL, KWL and AC wrote the first draft. All authors read and approved the final manuscript.

## Consent

Written informed consent was obtained from the patient for publication of this case report and accompanying images. A copy of the written consent is available for review by the Editor-in-Chief of this journal.
